# Prospective study to define the clinical utility and benefit of Decipher testing in men following prostatectomy

**DOI:** 10.1038/s41391-019-0185-7

**Published:** 2019-11-12

**Authors:** Joseph Marascio, Daniel E. Spratt, Jingbin Zhang, Edouard J. Trabulsi, Tiffany Le, Worlanyo Sosu Sedzorme, Whitney H. Beeler, Elai Davicioni, Bashar Dabbas, Daniel W. Lin, John L. Gore, Matthew Bloom, Mark Mann, J. Ryan Mark, Anne Calvaresi, James L. Godwin, Peter McCue, Mark D. Hurwitz, W. Kevin Kelly, Costas D. Lallas, Karen E. Knudsen, Leonard G. Gomella, Adam P. Dicker, Robert B. Den

**Affiliations:** 1 Department of Radiation Oncology, Thomas Jefferson University Hospital, Philadelphia, PA USA; 2 Department of Radiation Oncology, University of Michigan, Ann Arbor, MI USA; 3 Decipher Biosciences, San Diego, CA USA; 4 Department of Urology, Thomas Jefferson University Hospital, Philadelphia, PA USA; 5 Department of Urology, University of Washington, Seattle, WA USA; 6 Department of Medical Oncology, Thomas Jefferson University Hospital, Philadelphia, PA USA; 7 Department of Pathology, Thomas Jefferson University Hospital, Philadelphia, PA USA; 8 Sidney Kimmel Cancer Center, Philadelphia, PA USA

**Keywords:** Prognostic markers, Cancer therapy

## Abstract

**Background:**

Genomic classifiers (GC) have been shown to improve risk stratification post prostatectomy. However, their clinical benefit has not been prospectively demonstrated. We sought to determine the impact of GC testing on postoperative management in men with prostate cancer post prostatectomy.

**Methods:**

Two prospective registries of prostate cancer patients treated between 2014 and 2019 were included. All men underwent Decipher tumor testing for adverse features post prostatectomy (Decipher Biosciences, San Diego, CA). The clinical utility cohort, which measured the change in treatment decision-making, captured pre- and postgenomic treatment recommendations from urologists across diverse practice settings (*n* = 3455). The clinical benefit cohort, which examined the difference in outcome, was from a single academic institution whose tumor board predefined “best practices” based on GC results (*n* = 135).

**Results:**

In the clinical utility cohort, providers’ recommendations pregenomic testing were primarily observation (69%). GC testing changed recommendations for 39% of patients, translating to a number needed to test of 3 to change one treatment decision. In the clinical benefit cohort, 61% of patients had genomic high-risk tumors; those who received the recommended adjuvant radiation therapy (ART) had 2-year PSA recurrence of 3 vs. 25% for those who did not (HR 0.1 [95% CI 0.0–0.6], *p* = 0.013). For the genomic low/intermediate-risk patients, 93% followed recommendations for observation, with similar 2-year PSA recurrence rates compared with those who received ART (*p* = 0.93).

**Conclusions:**

The use of GC substantially altered treatment decision-making, with a number needed to test of only 3. Implementing best practices to routinely recommend ART for genomic-high patients led to larger than expected improvements in early biochemical endpoints, without jeopardizing outcomes for genomic-low/intermediate-risk patients.

## Introduction

The use of radical prostatectomy (RP) has increased in the management of high-risk prostate cancer (PCa) [1]. Current clinicopathologic nomograms estimate that 30–95% of these men may experience biochemical recurrence (BCR) within the first 5 years after surgery [2]. To help improve these outcomes, multiple randomized trials assessed the benefit of adjuvant radiotherapy (ART) after surgery for men with adverse pathologic (AP) features, including extracapsular extension, seminal vesicle involvement, or positive margins. In each of these trials ART improved biochemical progression-free survival [2–4]. Furthermore, the SWOG 8794 demonstrated improvements in overall survival (OS) [5]. Thus, it is probable that subsets of men with AP features may derive large benefits from the use of ART, while other men derive minimal benefit.

Given that the ART-randomized trials also demonstrated a small, but consistent, increase in grade ≥3 toxicity from the addition of ART, the use of ART for all men with AP has not been adopted in most of the world. In the United States, despite consistent guideline endorsement for discussion of ART for men with AP, approximately only 10% of men with AP features receive ART, and many practices never utilize ART regardless of clinicopathologic risk estimates of recurrence [6–8]. It is probable that there are men harboring potentially lethal PCa that would be undertreated with surgical monotherapy.

The use of ART remains low given that current clinicopathologic systems have a moderate ability to identify which patients will have biologically aggressive disease and will derive maximal benefit from treatment intensification. Fortunately, gene expression classifiers, such as the Decipher RP test, have been shown to more accurately risk stratify prostatectomy patients, and identify patients that will derive the most benefit from ART as opposed to salvage radiation therapy (SRT) [9, 10]. This genomic classifier (GC) test has been validated in over 3000 patients, and has been shown to be prognostic in a randomized clinical trial [11]. As well, the incorporation of genomics improves patient decision-making and reduces patient anxiety with postoperative decisions, as demonstrated in a prospective trial [12].

Based on these findings, we implemented a prospective registry imbedded within the Sidney Kimmel Cancer Center at Thomas Jefferson University (TJU) multidisciplinary tumor board based on the genomic-risk score of a patient. Those with low- and intermediate-risk scores would be recommended for observation postoperatively, and high-risk patients would be recommended for ART. We also present an analysis of a large prospective cohort from the Medicare Decipher testing registry, which requires the manufacturer to track adverse events, treatment decisions and utilization of GC clinical testing for Medicare beneficiaries. We hypothesized that GC-high-risk patients that followed the tumor board recommendation would have improved biochemical control over those that deviated from these prespecified recommendations, with no detriment to those with low GC scores.

## Patients and methods

### Study cohorts

This study included two prospective observational cohorts of men diagnosed with PCa and treated with RP. The clinical utility (CU) cohort is from the Medicare registry that the test manufacturer (Decipher Biosciences, San Diego, CA) is obligated by CMS to maintain in order to track GC-based treatment recommendation changes from ordering physicians as a requirement for providing the test to Medicare beneficiaries. The second is a clinical benefit (CB) cohort from TJU that implemented GC-based treatment recommendations and tracked patients systematically for early BCR.

The CU cohort consisted of 3910 patients tested with Decipher by providers enrolled in the Medicare Certification and Training registry (CTR), which is maintained by the manufacturer and administered by the Medicare Administrative Contractor Palmetto GBA’s Molecular Diagnostics (“MolDX”) program (Columbia, SC). Between October 2016 and January 2019, 823 providers using GC were enrolled in the CTR. All providers received mandatory training on the appropriate utilization of the test required to indicate treatment recommendations for their patients. Providers submitted management recommendations upon ordering the GC test and again upon receiving the test results. Only Medicare patients that met the adjuvant setting local coverage determinations inclusion criteria (pathological stage ≥pT3 or positive margins) and whose provider was certified in the CTR registry were included in the analysis. Details of the MolDX program can be found here and the data collection instruments are available online. All the CTR patient-related data were deidentified and study researchers were blinded to patient identifiers.

Institutional review board approval was obtained for the CB cohort from TJU prior to initiation of the study. Patients were all treated by RP between March 2014 and August 2016. Eligible patients for this study were required to have undetectable PSA after RP and harbor one or more AP features (positive surgical margins or pT3 disease). Patients were excluded if they harbored lymph node positive or metastatic disease at diagnosis or if they had received neoadjuvant therapy.

### Specimen collection, handling, and GC testing

Tumor sample was selected from the formalin-fixed paraffin-embedded RP specimen with the highest Gleason score and tumor volume and sent for GC testing in a CAP/CLIA laboratory (Decipher Biosciences, San Diego, CA). A whole-transcriptome microarray assay, which measures the expression of over 46,000 genes and noncoding RNA, was performed as previously described [13]. Test results included the GC score (a 22-gene expression random forest model) on a continuous scale (0–1) and classified by genomic-risk groups (low, intermediate, or high) [14].

### Tumor board treatment recommendations

Starting in January 2014, a multidisciplinary meeting at TJU was held after review of the available validation data for the GC test. Given the ability of the GC test to more accurately identify men at high risk of recurrence, and those that would derive clinically meaningfully large benefits from ART, it was agreed upon that all RP patients with AP undergoing the GC test would be given personalized decision recommendations as follows: genomic low/intermediate-risk patients would be recommended for observation, and genomic high-risk patients would be recommended for ART.

### Endpoints

The primary endpoint was a priori chosen as early BCR within 2-year post-RP based on following the GC-based treatment recommendations. BCR was defined as PSA ≥ 0.2 ng/mL after achieving nadir (<0.1 ng/ml). Time to BCR was calculated from time of definitive treatment (either RP or ART if given) until event or last follow-up. This endpoint was selected based on recent evidence that has demonstrated that early BCR is a potential surrogate endpoint for both distant metastasis, PCa-specific mortality, and OS [15, 16]. In addition, the influence of the GC test on treatment decision-making in adjuvant setting is reported. Treatment recommendations were grouped into observation with PSA-monitoring, ART, ART with ADT, adjuvant ADT alone, and other.

### Statistical analysis

Descriptive statistics of the two cohorts are reported by medians and interquartile-ranges (IQR) or frequencies and proportions, as appropriate. Fisher’s exact test was used to compare treatment recommendations across GC risk groups. Cumulative incidence curves of PSA recurrence risk were constructed and compared using log-rank test. Univariable and multivariable logistic regression models were used to study the association between risk variables and treatment decision-making. Univariable and multivariable Cox proportional hazard models were used to assess BCR. Statistical analyses were performed in R V3.0 (R Foundation, Vienna, Austria) and all statistical tests were two-sided, using a 5% significance level.

## Results

### CU cohort characteristics

Between October 2016 and January 2019 (CTR reporting period), 3910 patients were enrolled: 3455 (88%) in the adjuvant (PSA < 0.1 ng/ml) and 455 (12%) salvage (PSA > 0.1 ng/ml) setting. Demographic and pathological characteristics of the CU adjuvant cohort are provided in Table 1. GC classified 28, 24, and 48% as low- (GC < 0.45), intermediate- (0.45–0.60), and high- (>0.60) genomic-risk, respectively. These correspond to a 5-year metastasis rate of < 4%, 4–9% and > 9%, respectively. Providers in the CTR were from large urology group practice (LUGPA, 40%), community (31%), and academic (20%) practice settings. Enrolled physicians in the registry providing treatment recommendations were primarily urologists (95%).

**Table 1:** Baseline characteristics of the prospective cohorts

Variables	Clinical utility	Clinical benefit
No. patients, *n* (%)	3455 (97.1)	102 (3.1)
Age		
Median (range)	69 (43, 89; NA = 5)	63.3 (43.8, 73.9)
Preoperative PSA (ng/mL)		
Median (range)	6.9 (0.008, 131; NA = 1491)	6.08 (2.3, 46)
RP grade group, *n* (%)		
GG 1	106 (3.1)	3 (2.9)
GG 2	1259 (36.4)	42 (41.2)
GG 3	1156 (33.5)	37 (36.3)
GG 4	349 (10.1)	9 (8.8)
GG 5	585 (16.9)	11 (10.8)
Pathological T stage, *n* (%)		
pT2	749 (21.7)	12 (11.8)
pT3a	1727 (50.0)	71 (69.6)
pT3b	979 (28.3)	19 (18.6)
Positive surgical margins, *n* (%)		
No	1376 (39.8)	56 (54.9)
Yes	2079 (60.2)	46 (45.1)
GC risk group, *n* (%)		
High risk	1674 (48.5)	62 (60.8)
Intermediate risk	819 (23.7)	16 (15.7)
Low risk	962 (27.8)	24 (23.5)
Follow-up time, months		
Median (Range)	Unavailable	22 (3, 52)

*GC* genomic classifiers*, GG* grade group*, No.* number*, PSA* prostate-specific antigen*, RP* radical prostatectomy

### Provider treatment recommendations

Among enrolled patients, 2002 (58%) had both pre- and post-GC provider treatment recommendations available for analysis. Pre-GC in the CU cohort, providers recommended observation with PSA-monitoring for 69% (*n* = 1384), ART for 25% (*n* = 501), ART + ADT for 5% (*n* = 92), and adjuvant ADT alone for 1% (*n* = 25) of the patients.

In the CU cohort pre-GC, observation with PSA-monitoring was recommended for 78% (*n* = 757) of patients treated by providers in the community, 67% (*n* = 460) in academic, and 60% (*n* = 765) in LUGPA practice settings (Fig. S1A). Urologists recommended observation with PSA-monitoring for 69% (*n* = 2026) compared with 54% (*n* = 29) of patients by radiation/medical oncologists in the adjuvant cohort (Fig. S1B).

Post-GC, provider treatment recommendations changed for 39% of patients in the CU cohort. Overall, treatment was intensified or de-intensified for 18 and 21% of adjuvant cases, and 30 and 14% of salvage cases. Observation with PSA-monitoring increased to 75%, ART decreased to 14%, and ART + ADT and ADT alone increased to 9 and 2% (*p* < 0.001). While accounting for individual clinical-risk factors or the Cancer of the Prostate Risk Assessment Score (CAPRA-S), higher genomic-risk was significantly associated with intensification of therapy (Table 2).

**Table 2:** Univariable (UVA) and multivariable (MVA) logistic regression model for prediction of treatment recommendation for clinical utility cohort

		UVA		MVA	
Variable	Category	OR (95% CI)	*P* value	OR (95% CI)	*P* value
Age		1.0 (1.0–1.0)	0.563	1.0 (0.9–1.0)	0.006*
PSA		1.0 (1.0–1.0)	0.065	1.0 (1.0–1.0)	0.519
Pathological GG	3 vs 1–2	2.5 (2.0–3.3)	<0.001*	2.1 (1.4–2.9)	<0.001*
	4–5 vs 1–2	3.5 (2.7–4.5)	<0.001*	1.8 (1.2–2.6)	0.003*
Pathological T stage	pT3a vs pT2	1.4 (1.1–1.9)	0.013*	1.3 (0.8–2.0)	0.251
	pT3b vs pT2	2.8 (2.1–3.8)	<0.001*	1.7 (1.1–2.7)	0.030*
SM	Yes vs no	1.2 (1.0–1.5)	0.056	1.4 (1.0–2.0)	0.024*
GC risk group	Intermediate vs low	2.5 (1.7–3.8)	<0.001*	1.9 (1.1–3.3)	0.024*
	High vs low	9.5 (6.8–13.3)	<0.001*	8.5 (5.3–13.6)	<0.001*
CAPRA-S	3–5 vs 0–2	4.3 (1.5–12.1)	0.005*	3.9 (1.4–11.2)	0.011*
	6–12 vs 0–2	8.8 (3.2–24.6)	<0.001*	6.2 (2.1–17.6)	<0.001*
GC risk group	Intermediate vs low	2.5 (1.7–3.8)	<0.001*	1.9 (1.1–3.4)	0.019*
	High vs low	9.5 (6.8–13.3)	<0.001*	8.7 (5.4–13.8)	<0.001*

*CAPRA-S* Cancer of the Prostate Risk Assessment score, *GC* genomic classifiers, *GG* grade group, *MVA* multivariable analysis, *OR* odds ratio, *PSA* prostate-specific antigen, *SM* surgical margin, *UVA* univariable analysis, *95% CI* 95% confidence interval, *significant *p*-value

**Fig. 1:**
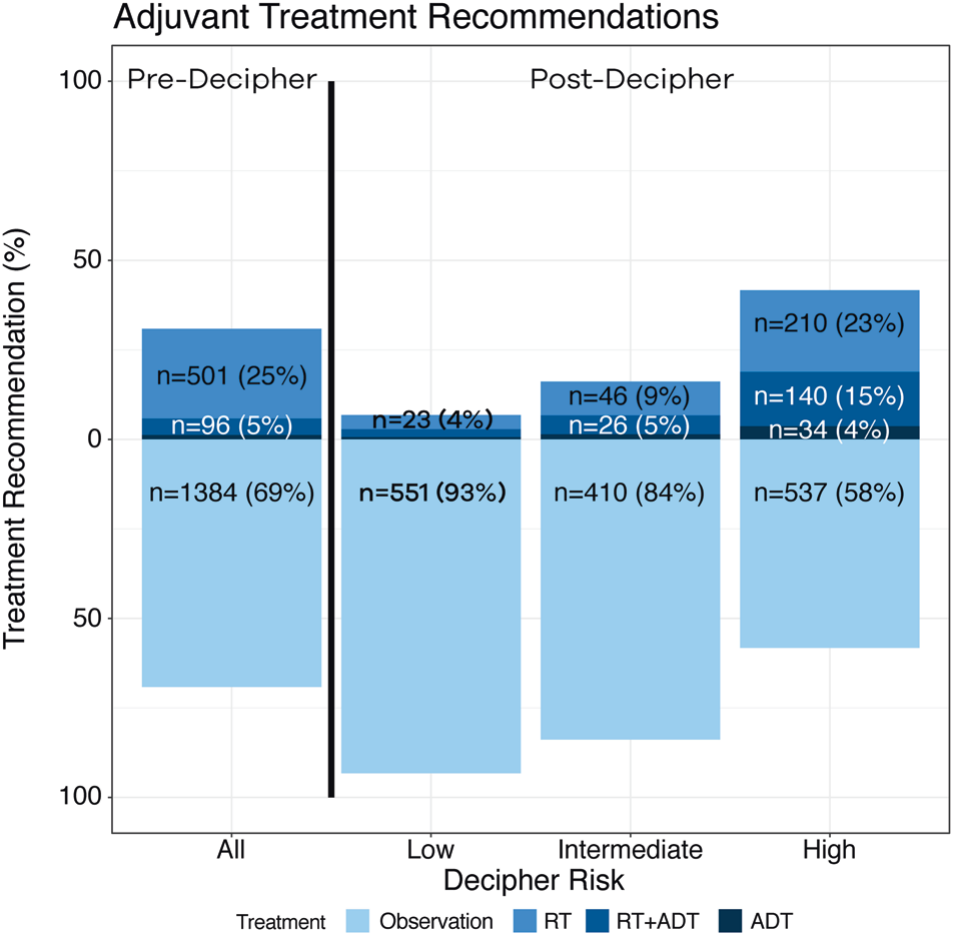
Treatment recommendation bar plot for clinical utility cohort, demonstrating recommendations before and following genomic classifier testing. ADT androgen deprivation therapy, RT radiotherapy

When stratified by genomic-risk (Fig. 1) in the CU cohort, observation with PSA monitoring was recommended for 93, 84, and 58% of low, intermediate, and high genomic-risk patients (*p* < 0.001).

### CB cohort

Providers and patients were recommended to adhere to, but not mandated to follow, the tumor board best practice. From March 2014 through August 2016, GC was ordered for 135 patients considering adjuvant therapy after RP. Nineteen patients with detectable PSA, one patient with positive lymph nodes and one patient with BCR prior to ordering GC testing were excluded from study. In addition, four patients with incomplete pathologic information and six patients with missing follow-up information were excluded, leaving 102 patients for analysis.

Demographic and pathologic characteristics of CB cohort are listed in Table 1. The median age at RP was 64 with a median follow-up of 22.5 months. In total 88% of patients were pT3a or greater and 45% had positive surgical margins. Based on the tumor board best practice recommendations, ART was recommended for 61% of patients. The best practice was followed for 74% of patients; a similar adherence rate to physician recommendations as observed in a recent prospective study [12].

**Fig. 2:**
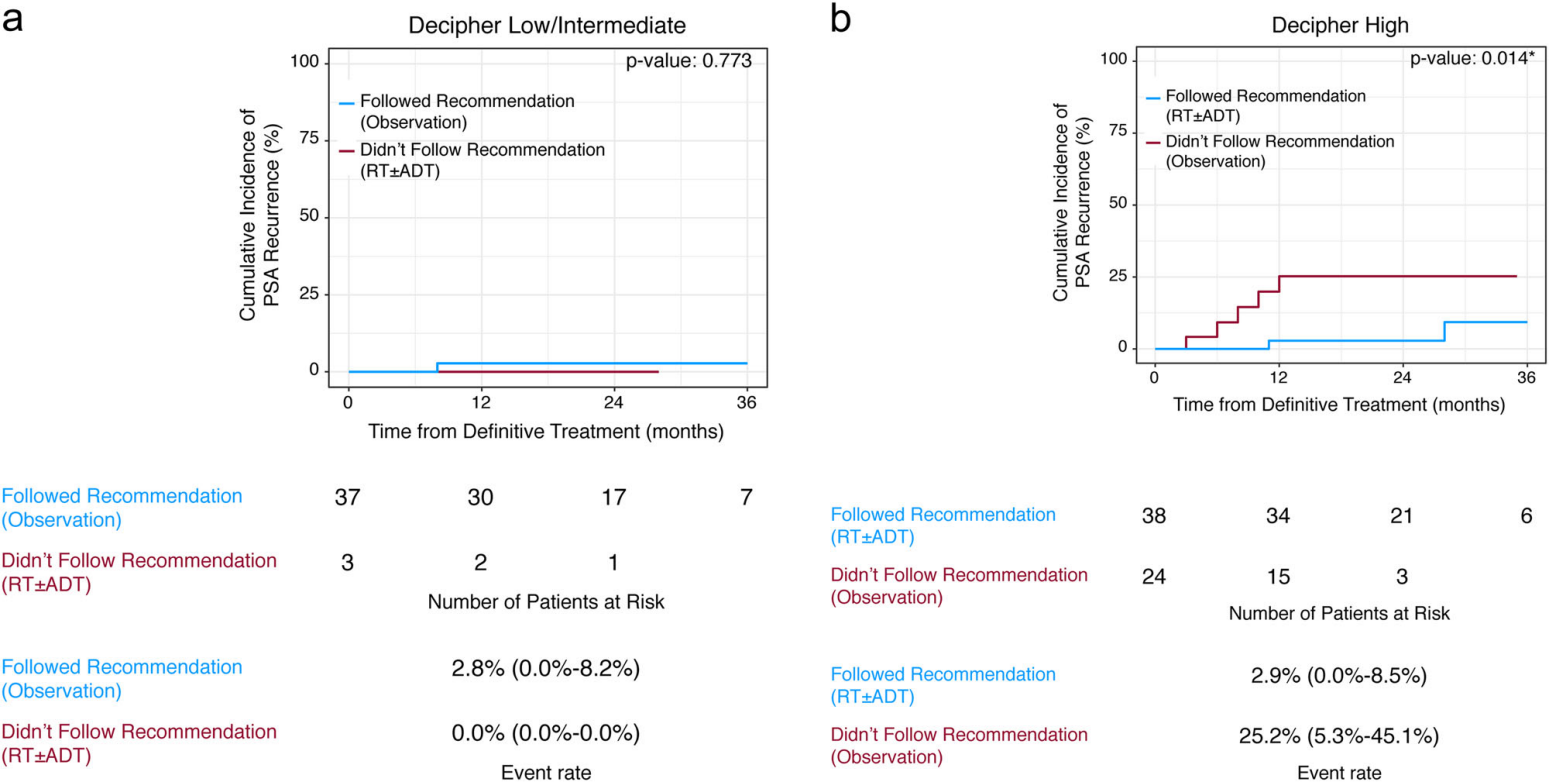
Cumulative incidence plot stratified by compliance with tumor board treatment recommendation for **a** genomic-low/intermediate-risk patients, and **b** genomic-high-risk patients. ADT androgen deprivation therapy, PSA prostate-specific antigen, RT radiotherapy

Two-year cumulative incidence of PSA recurrence was 3% for those that followed the tumor board recommendation compared with 22% for those that did not (*p* = 0.004). For the low- and intermediate-GC risk patients, 93% followed the recommendation for observation with PSA-monitoring. 2-year PSA recurrence for those who followed the recommendation was 3% vs 0.5% for those that received ART (Fig. 2a, *p* = 0.77). For patients with high-GC risk that followed the recommendation for ART versus those that did not, the 2-year cumulative incidence of PSA recurrence was 3% vs 25%, respectively (Fig. 2b, *p* = 0.01). While adjusting for CAPRA-S, those who followed recommendation for ART had significantly lower risk of PSA recurrence (Table 3, HR = 0.1 [0.0–0.6], *p* = 0.013). Importantly, given the efficacy of ART and its near exclusive use in high-genomic-risk patients, differences in BCR were no longer significant when patients were stratified by GC risk group (*p* = 0.1).

**Table 3:** Univariable (UVA) and multivariable (MVA) Cox regression model for prediction of early biochemical recurrence for clinical benefit cohort

		UVA		MVA	
GC Subset	Variable	HR (95% CI)	*P* value	HR (95% CI)	*P* value
Low/intermediate risk	CAPRA-S	1.5 (0.5–2.7)	0.345	1.4 (0.0–1265.0)	0.449
	Treated vs not treated	4.0 (0.0–75.0)	0.456	1.3 (0.0–84.3)	0.926
High risk	CAPRA-S	1.2 (0.8–1.8)	0.430	1.5 (0.9–2.3)	0.093
	Treated vs not treated	0.2 (0.0–0.8)	0.030*	0.1 (0.0–0.6)	0.013*

*CAPRA-S* Cancer of the Prostate Risk Assessment score, *GC* genomic classifiers, *HR* hazard ratio, *MVA* multivariable analysis, *PSA* prostate-specific antigen, *UVA* univariable analysis, *95% CI* 95% confidence interval, *significant *p*-value

## Discussion

Three randomized prospective trials comparing ART with observation in men with AP features following RP have demonstrated improvements in biochemical progression-free survival at the expense of a slight increase in severe toxicity. In addition, approximately half of the men randomized to observation did not experience BCR at 5 years post-RP. Even those with biochemical failure can have varied outcomes, with nearly 50% of patients remaining free of metastatic disease at 10 years without any further treatments [17]. The clinical conundrum is selecting which patients would benefit from early intervention, and in those, who could continue with observation. This is important, as the SWOG 8794 randomized trial demonstrated an OS benefit with postoperative RT, implying that, in the appropriately selected patient population, ART will improve survival outcomes.

Multiple tools and nomograms have been created in an effort to predict failure post-RP. Postoperative nomograms include the CAPRA-S and Stephenson nomograms, which rely on clinicopathologic factors such as preoperative PSA, Gleason score, staging, and surgical margins [18–20]. Spratt et al. has demonstrated, in an individual patient meta-analysis of nearly 1000 patients, that across all patient population undergoing RP, the GC adds independent prognostic information above and beyond multivariable models for the prediction of metastatic disease [21]. Furthermore, the ability to more accurately risk stratify patients has translated into the ability to better identify patients with sufficient risk of recurrence to benefit from ART [9, 10]. This functionality has led to its support in the NCCN guidelines with a defined role following prostatectomy [22]. In addition to a lower rate of metastases, lower GC risk scores also predict for a longer time interval to metastasis [9]. This lends itself to a surveillance and salvage approach in low-risk GC patients.

The PRO-IMPACT prospective trial has demonstrated that GC testing post-RP favorably impacts treatment decision-making post-RP, promoting more postoperative radiotherapy for those with GC high risk and more observation for those with GC-low/intermediate risk. This translated not only into prospective favorable CU, but also demonstrated significantly improved patient reported quality of life, a recognized metric of CB [12]. In this report, we confirm these findings in a much larger and more diverse patient and provider population through the Medicare CTR. This consists of various specialties that ordered the GC test, and diverse urology practice types. We show very similar rates of GC testing impacting treatment decisions, with the PRO-IMPACT study having a number needed to test of 4, and the CTR cohort having a number needed to test of 3. Importantly, both studies did not prespecify how providers should interpret or act upon the results. Thus, with further understanding of the optimal use of GC testing it is probable that the number needed to test to change one treatment decision will continue to further be reduced.

Contemporary work has consistently shown that only ~10% of urologists recommend ART for men with AP. This is very concerning given that many high-risk men have a >75% chance of recurring within 5 years post-RP as predicted by Memorial Sloan Kettering nomograms. We show that urologists are very receptive to using GC testing to personalize the use of ART and are ninefold more likely to recommend ART for GC-high-risk patients. Importantly, there is not an overall increase in the use of ART given that nearly all GC-low/intermediate risk patients are recommended observation.

A common criticism of CU studies is that it is unclear if the treatment decisions being made favorable impact tumor control outcomes or quality of life. The PRO-IMPACT trial already demonstrated a quality of life benefit for GC testing, and here we provide evidence for oncologic benefit as well. A systematic and agreed upon multidisciplinary tumor board approach to define “best practice” of routine recommending of ART for GC high-risk patients demonstrated that ~75% of them now received ART, in contrast to population estimates of 10%. For the GC high patients that received ART, this translates to a tenfold reduction in the risk of early BCR (HR 0.1). This should be compared with that from the unselected populations in the three randomized trials which had a HR of 0.5. Thus, not only does GC testing select those patients are most likely to recur, it also potentially identifies patients most likely to benefit from ART, as shown in previous reports.

Early BCR has repeatedly been shown to be a potential surrogate endpoint for OS in multiple-randomized trials [15, 16]. It should be recognized that no prognostic tool in PCa has demonstrated an impact on OS. For example, the wide-spread adoption of MRI technology is based on trials demonstrating the ability of MRI to identify more clinically significant PCa lesions. Similarly, molecular PET imaging, which has gained FDA approval has only demonstrated CU to change management without associated CB. There are no prospective studies for either technology that demonstrate a favorable impact on early BCR, let alone death from PCa.

This study is not without limitations. In the CTR cohort, as previously discussed, providers and patients were not advised to “best practices” based on GC test results. However, this is also a strength in that it provides real-world estimates of the impact of GC testing in practice. Our study reported on time to early BCR, and further independent validation is always recommended. Additional follow-up may detect differences in other longer-term endpoints. The randomized trial G-MINOR (NCT02783950), which recently completed accrual, will build upon this data, which randomizes patients to GC testing or best available standard of care (CAPRA-S) for patients with adverse pathology to further establish the CU and CB of GC testing.

## Conclusions

The GC has previously established analytical and clinical validation with superior prognostic performance over clinicopathologic multivariable models. We now validate the previous PRO-IMPACT trial results in a Medicare prospective registry demonstrating favorable impact on CU and postoperative treatment recommendations. The use of GC substantially altered treatment decision-making, with a number needed to test of only 3. Implementing best practices to routinely recommend ART for genomic-high patients led to larger than expected improvements in early biochemical endpoints, without jeopardizing outcomes for genomic low/intermediate-risk patients. This data should support inclusion of GC directed utilization of ART in clinical practice guidelines.

## Supplementary information

Supplementary Figure S1A

Supplementary Figure S1B
