# Supplementary figures and images for: Prospective study to define the clinical utility and benefit of Decipher testing in men following prostatectomy
Source: Prostate Cancer Prostatic Dis. 2019 Nov 12;23(2):295–302. doi: 10.1038/s41391-019-0185-7 (PMC7237345; doi:10.1038/s41391-019-0185-7)

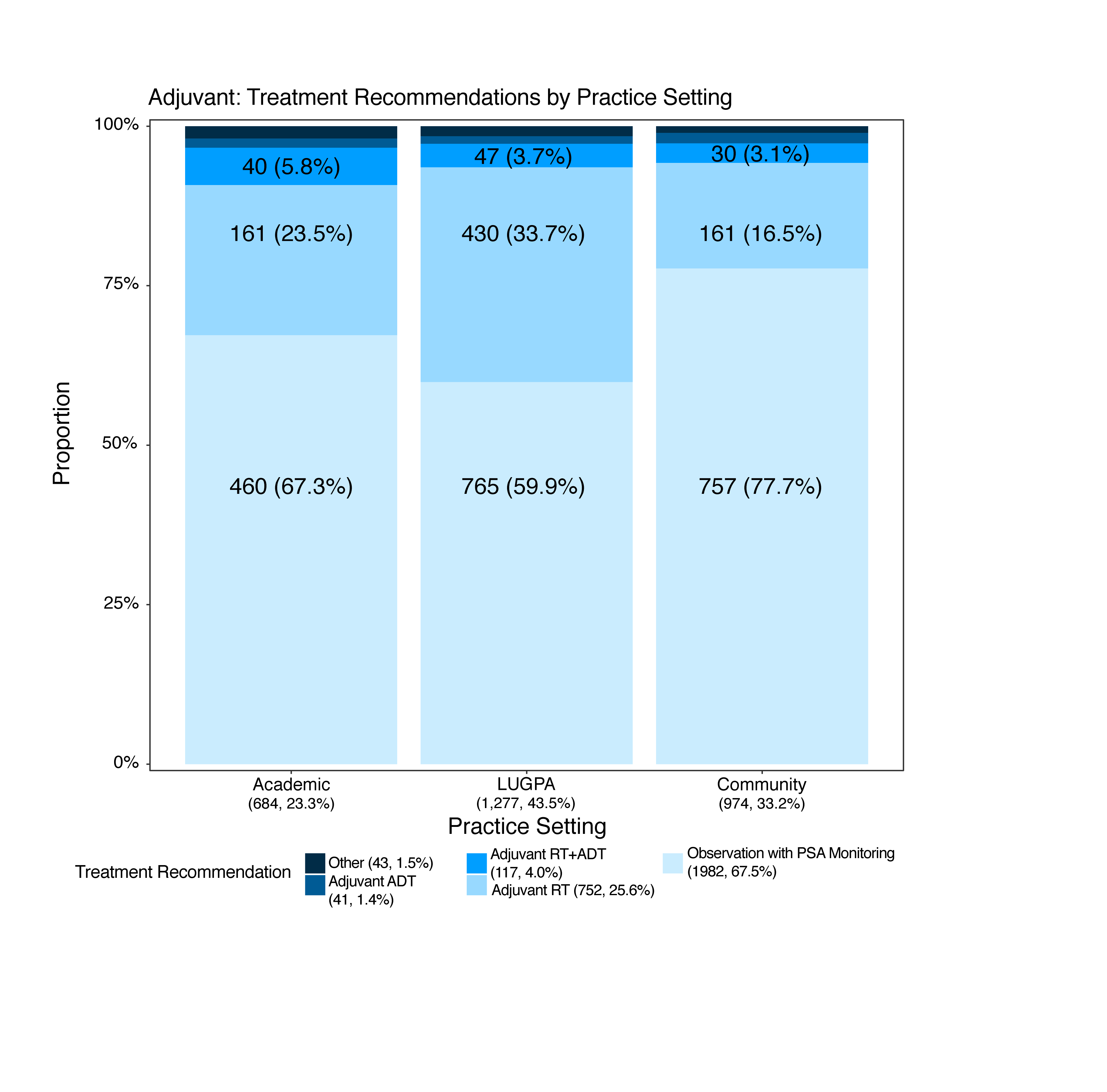

Supplement: Supplementary file 1 — Supplementary Figure S1A [file 41391_2019_185_MOESM1_ESM.tif]

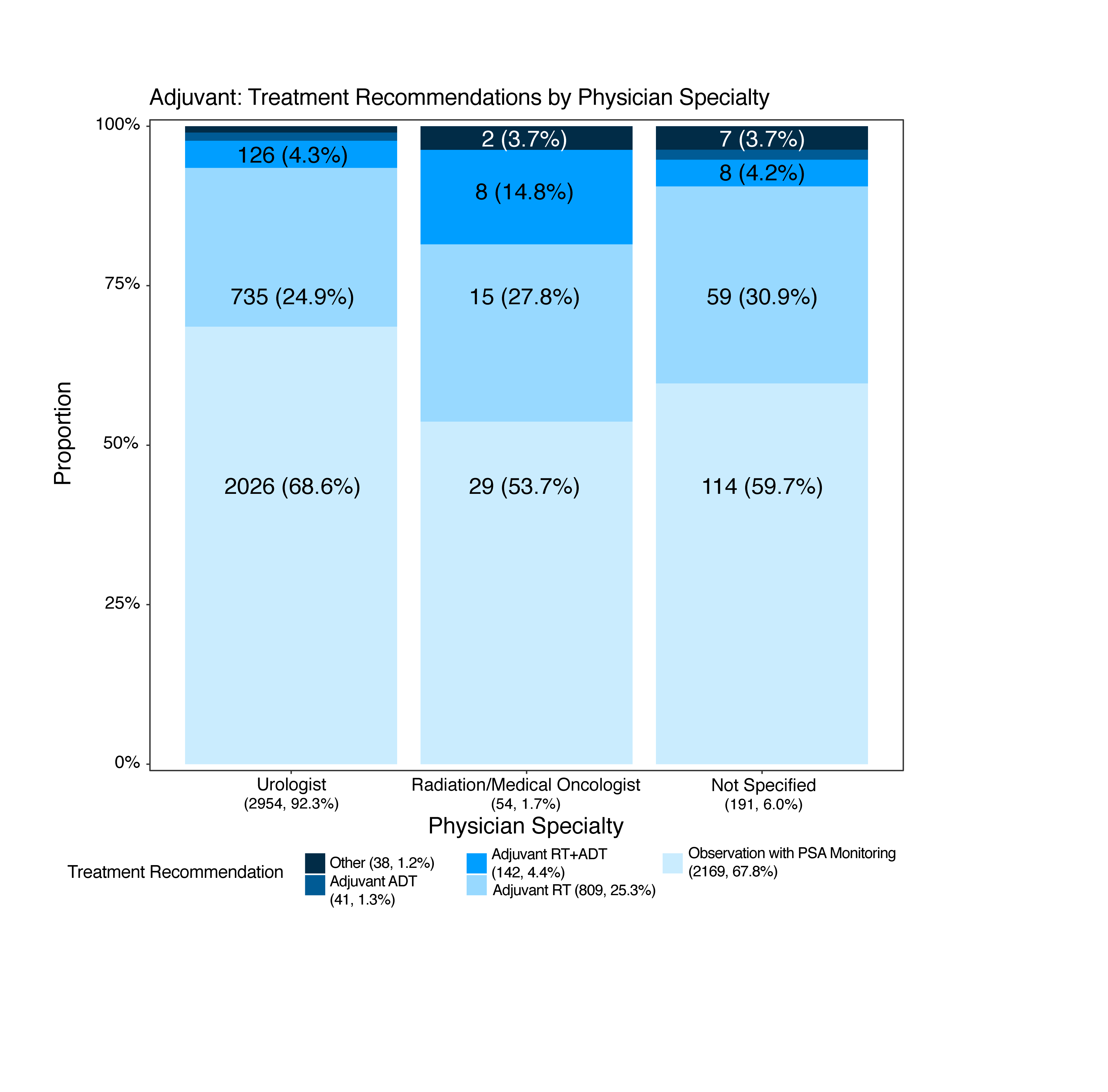

Supplement: Supplementary file 2 — Supplementary Figure S1B [file 41391_2019_185_MOESM2_ESM.tif]
